# Association of postoperative anxiety and depressive symptom severity with progression-free survival in IDH-wild-type glioblastoma

**DOI:** 10.3389/fonc.2026.1949803

**Published:** 2026-10-19

**Authors:** Zhang Xiong, Zixiao Li, Changli Han, Xiangxiang Peng

**Affiliations:** 1 Department of Neurosurgery, The First Affiliated Hospital of Wannan Medical University, Wuhu, Anhui, China; 2 Institutes of Brain Science, Wannan Medical University, Wuhu, Anhui, China; 3 Department of Neurology, The First Affiliated Hospital of Wannan Medical University, Wuhu, Anhui, China

**Keywords:** anxiety, depression, GAD-7, glioblastoma, IDH-wild-type, PHQ-9, progression-free survival, transcriptomics

## Abstract

**Background:**

Anxiety and depressive symptoms are common in patients with glioblastoma, but their association with progression-free survival (PFS) remains incompletely characterized in molecularly defined populations. We examined these associations in patients with IDH-wild-type glioblastoma.

**Methods:**

This single-center observational study included 64 adults with newly diagnosed IDH-wild-type glioblastoma who completed the Generalized Anxiety Disorder-7 (GAD-7) and Patient Health Questionnaire-9 (PHQ-9) at a median of 35 days after resection (range, 30–38 days), before postoperative adjuvant therapy. Scores ≥10 defined positive screens. Separate multivariable Cox models estimated associations with PFS, adjusting for age, Karnofsky Performance Status, and gross-total resection. An independent exploratory analysis compared neurotransmitter- and stress-related receptor expression in paired primary and recurrent tumors from 105 patients annotated as having IDH-wild-type glioblastoma in the Glioma Longitudinal Analysis Consortium (GLASS).

**Results:**

Compared with scores below the respective screening thresholds, greater anxiety symptom burden [GAD-7 score ≥10; adjusted hazard ratio 1.92, 95% confidence interval (CI) 1.08–3.42] and greater depressive symptom burden (PHQ-9 score ≥10; adjusted hazard ratio 4.22, 95% CI 1.72–10.35) were associated with shorter PFS. Supportive continuous-score analyses showed consistent associations. In the independent GLASS cohort, recurrent tumors showed higher adrenergic, serotonergic, dopaminergic, and GABAergic receptor-family scores, higher *CRHR1* expression, and lower *AVPR1A* expression.

**Conclusion:**

Greater postoperative anxiety and depressive symptom burden was associated with shorter PFS in IDH-wild-type glioblastoma. Prospective studies with repeated symptom assessments, more complete clinical data, and matched molecular sampling are needed to evaluate these observational associations and the separate, hypothesis-generating molecular findings.

## 1 Introduction

Glioblastoma, IDH-wild-type, CNS WHO grade 4, is an aggressive adult-type diffuse glioma defined in the fifth edition of the World Health Organization Classification of Tumors of the Central Nervous System (WHO CNS5) (1). Standard treatment consists of maximal safe resection followed by radiotherapy with concurrent temozolomide and then adjuvant temozolomide, commonly termed the Stupp regimen (2, 3). Nevertheless, most tumors progress and long-term disease control remains uncommon. This clinical course places substantial demands on patients as they recover from surgery and prepare for further treatment.

During the postoperative period, patients may face neurological deficits, loss of independence, uncertainty about prognosis, and fear of disease progression. Anxiety and depressive symptoms are common in glioma, including before postoperative radiotherapy and temozolomide, and are associated with poorer quality of life and greater supportive-care needs (4, 5). Clinical practice guidelines therefore recommend systematic assessment and management of these symptoms in cancer care (6, 7).

Whether postoperative anxiety and depressive symptom burden is associated with earlier tumor progression remains unclear. A 2024 meta-analysis found that anxiety and depression were associated with shorter overall survival in glioma, and several glioblastoma-specific or postoperative glioma studies reported similar findings (8–10). However, previous studies combined different tumor grades and molecular diagnoses, used different screening instruments and assessment times, and focused mainly on overall survival. Evidence is particularly limited for progression-free survival (PFS) when anxiety and depression are assessed during early postoperative recovery in patients with WHO CNS5-defined, IDH-wild-type glioblastoma.

Experimental studies of stress-responsive and neuronal signaling provide a rationale for exploring related receptor-gene expression in glioma (11, 12). Sympathetic catecholamine and hypothalamic–pituitary–adrenal pathways can alter tumor and immune biology (13, 14). In a murine prostate cancer model, psychosocial stress altered tumor inflammatory cytokines and accelerated tumor growth (15). Chronic stress accelerated experimental glioblastoma growth through dopamine–*DRD2* signaling (11), while neuronal neurotransmitter signaling can directly promote glioma progression (12). Paired primary and recurrent tumors therefore provide an opportunity to compare the expression of these receptor systems within the same patients at two disease stages.

We therefore examined whether anxiety and depressive symptoms assessed using Generalized Anxiety Disorder-7 (GAD-7) and Patient Health Questionnaire-9 (PHQ-9) during postoperative recovery, before the initiation of adjuvant therapy, were associated with PFS in adults with IDH-wild-type glioblastoma. Separately, we conducted an exploratory analysis of neurotransmitter- and stress-related receptor expression in paired primary and recurrent tumors from Glioma Longitudinal Analysis Consortium (GLASS). This analysis aimed to describe expression differences across disease stages and generate hypotheses for future studies with linked symptom and molecular measurements.

## 2 Materials and methods

### 2.1 Study design and participants

This single-center cohort combined prospectively collected symptom-screening data with retrospective clinical and follow-up information and followed the Strengthening the Reporting of Observational Studies in Epidemiology statement (16). We assessed patients who underwent neurosurgery at our institution between January 2024 and December 2025 and received a postoperative integrated diagnosis of glioblastoma, IDH-wild-type, CNS WHO grade 4 according to WHO CNS5 (1). IDH status was established through the institutional molecular and immunohistochemical workflow before symptom assessment.

Eligible patients were adults who underwent an index resection, completed both GAD-7 and PHQ-9 at the postoperative follow-up visit before initiation of adjuvant therapy, and subsequently received radiotherapy with concurrent temozolomide. Assessments were performed 30–38 days after surgery (median, 35 days). Exclusion criteria were reoperation for recurrent glioblastoma, age <18 years, impaired consciousness at assessment, biopsy only, or refusal of postoperative concurrent chemoradiotherapy (**Figure 1A**).

**Figure 1:**
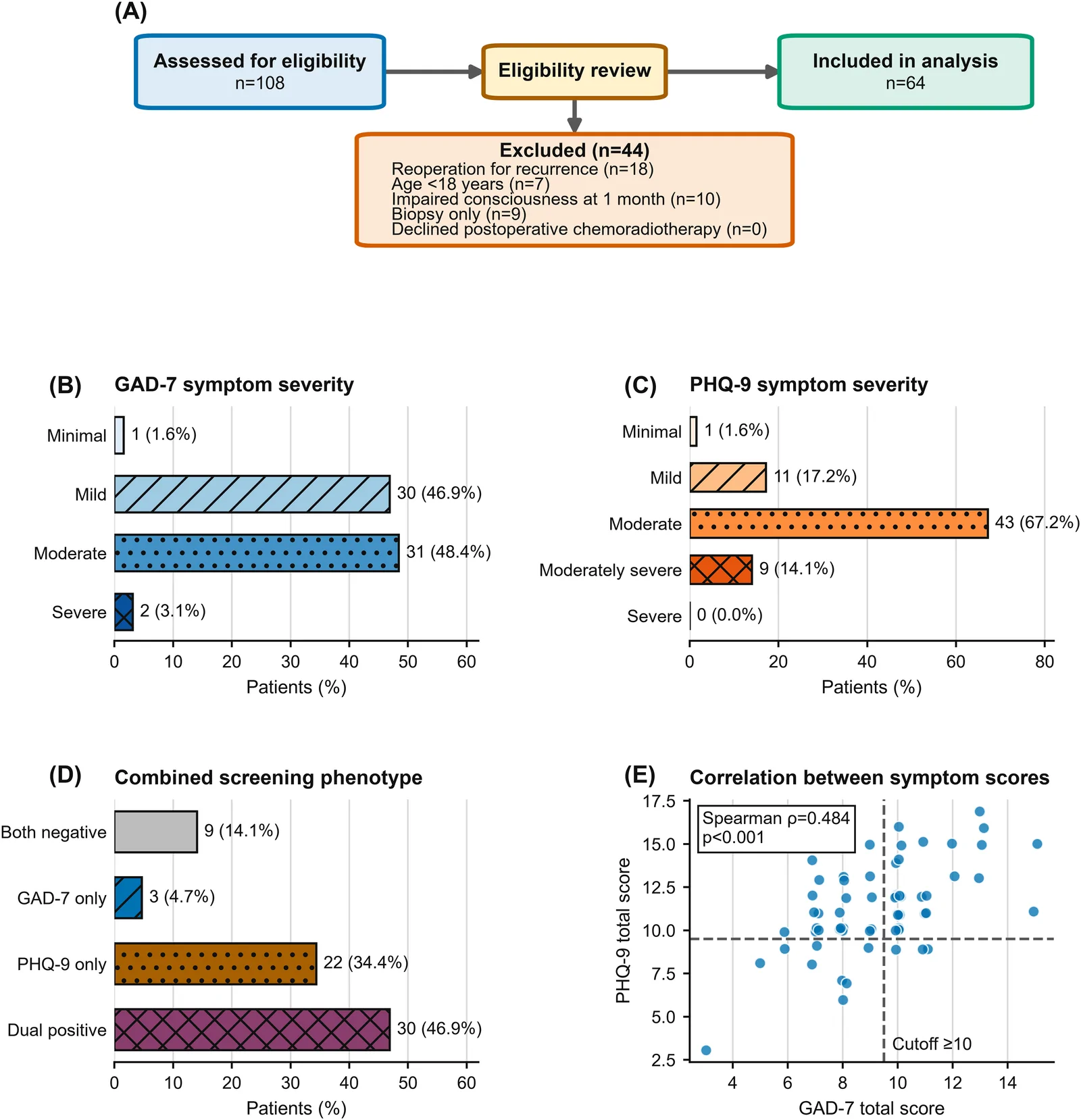
Participant flow and postoperative symptom-screening profile before postoperative adjuvant therapy. Symptom assessment occurred 30–38 days after surgery (median, 35 days). **(A)** Of 108 patients assessed for eligibility, 44 were excluded because of reoperation for recurrent glioblastoma (*n* = 18), age <18 years (*n* = 7), impaired consciousness at the postoperative assessment (*n* = 10), biopsy only (*n* = 9), or declining postoperative concurrent chemoradiotherapy (*n* = 0); 64 patients were included. **(B)** GAD-7 severity distribution. **(C)** PHQ-9 severity distribution. **(D)** Combined GAD-7/PHQ-9 screening phenotypes. Percentages in panels B–D use *n* = 64 as the denominator. **(E)** Association between GAD-7 and PHQ-9 total scores. Points represent individual patients and are jittered to display overlapping observations. Dashed lines indicate the screen-positive threshold of 10; Spearman’s ρ and the two-sided *p*-value are shown.

Recorded variables included age, sex, Karnofsky Performance Status (KPS) at screening, preoperative magnetic resonance imaging (MRI) features, extent of resection, treatment, early postoperative complications, and follow-up. Gross-total resection (GTR) was classified from operative and postoperative imaging records. Concurrent chemoradiotherapy referred to radiotherapy with concurrent temozolomide as the concurrent phase of the Stupp regimen (2). All patients in this cohort received postoperative antiseizure medication.

The study was approved by the Ethics Committee of Wannan Medical University Hospital (approval: 2026267). All participants provided written informed consent. The study was conducted in accordance with the Declaration of Helsinki.

### 2.2 Symptom screening and outcome definition

GAD-7 (17) and PHQ-9 (18) assessed anxiety and depressive symptoms, respectively, at the routine postoperative visit before initiation of postoperative adjuvant therapy. Scores ≥10 defined screen positivity, indicating greater symptom burden rather than a diagnosis of an anxiety or depressive disorder. Conventional severity categories were used for descriptive analyses. Four combined screening phenotypes were defined: negative on both screens, GAD-7 positive only, PHQ-9 positive only, and dual positive.

PFS was selected as the measure of disease-control duration (19) and was defined as the interval from index surgery to radiographic progression or death from any cause, whichever occurred first. Progression was identified on serial contrast-enhanced MRI according to the Response Assessment in Neuro-Oncology (RANO) 2.0 criteria (19) and confirmed by the treating neuro-oncology team. Dates were abstracted retrospectively from the clinical records, without independent centralized radiological review. The eight patients without progression were confirmed alive and progression-free through the administrative data lock of 25 June 2026 and were censored on that date.

### 2.3 Paired GLASS transcriptomic analysis

Paired primary–recurrent RNA-sequencing profiles were obtained from the GLASS consortium (20). This cohort was independent of the institutional clinical cohort. GAD-7 and PHQ-9 scores were unavailable in the GLASS data used here, and matched transcriptomic measurements were unavailable for the clinical cohort; consequently, patient-level symptom–expression associations could not be examined. Both samples in each pair were required to have deposited annotations of glioblastoma and histological grade IV, with IDH-wild-type status recorded as IDH_STATUS = IDHwt or an IDH_CODEL_STATUS value beginning with IDHwt. Selection relied on these deposited histopathological and molecular annotations and did not involve independent WHO CNS5 reclassification. TCGA-associated cases were excluded. The final cohort included 105 patients with paired presentation and earliest available qualifying recurrent profiles: 96 first, 7 second, and 2 third recurrences. Cohort definitions and nested sensitivity subsets are detailed in **Supplementary Table S1**.

Ninety-four receptor genes were grouped into eight neurotransmitter families: cholinergic, dopaminergic, adrenergic, serotonergic, GABAergic, glycinergic, glutamatergic, and neuropeptide Y. To characterize the expression of selected stress-related receptor genes, an exploratory extension added *CRHR1*, *CRHR2*, *NR3C1*, *NR3C2*, *AVPR1A*, and *AVPR1B*, yielding a 100-gene panel. Kallisto transcripts-per-million (TPM) values (21) were transformed as log2(TPM + 0.5). For family-level analysis, the 94 neurotransmitter genes were standardized gene-wise across the 210 main-cohort profiles, and each family score was the mean of its member-gene *z* scores. The within-patient difference was calculated as recurrent minus primary.

Family-level and individual-gene differences were tested with two-sided Wilcoxon signed-rank tests. We report Hodges–Lehmann estimates with 2,000-resample patient-bootstrap 95% confidence intervals (CIs). Benjamini–Hochberg (BH) correction was applied across the eight family tests and separately across the 100 gene tests. Sensitivity analyses restricted the cohort by recurrence order and explicit IDH_STATUS annotation. Purity-adjusted ordinary least-squares regression used heteroskedasticity-consistent type 3 covariance and accounted for within-patient differences in tumor purity estimated with Estimation of STromal and Immune cells in MAlignant Tumor tissues using Expression data (ESTIMATE) (22). Detailed algorithms and software are provided in the **Supplementary Methods**.

### 2.4 Statistical analysis

Continuous variables are reported as median [interquartile range (IQR)] and categorical variables are reported as *n*/*N* (%), with available-case denominators. Spearman’s ρ assessed score correlation, and PFS was summarized using Kaplan–Meier curves and log-rank tests. Univariable Cox models estimated unadjusted hazard ratios (HRs). Separate multivariable Cox proportional hazards models were fitted for GAD-7 positivity, PHQ-9 positivity, dual positivity, and each standardized symptom score; every model simultaneously included the symptom measure, age, KPS, and GTR. Age and KPS were initially entered as linear continuous terms. Continuous symptom-score HRs are expressed per one sample standard deviation. GAD-7 and PHQ-9 screen positivity were the primary clinical comparisons, evaluated in separate models. The dual-positivity analysis was exploratory, and continuous-score analyses were supportive. The four-category screening phenotype was summarized descriptively because of small subgroup sizes, without formal hypothesis testing across the four categories.

Proportional hazards were assessed using scaled Schoenfeld residual tests and plots, including covariate-specific and global tests. Linearity of age, KPS, and continuous symptom scores were assessed using likelihood ratio tests comparing linear terms with two-degree-of-freedom natural cubic splines. To address nonproportionality for GTR, sensitivity analyses used GTR-stratified Cox models adjusted for age and KPS. Tests were two-sided with 95% CIs. Clinical analyses were performed in Python 3.11.15 using NumPy 2.4.6, pandas 3.0.3, and SciPy 1.17.1. Model diagnostics and sensitivity analyses used R 4.6.0 and the survival package, version 3.8.6. Further details are provided in **Supplementary Tables S5, S6** and **Supplementary Figures S4–S6**.

## 3 Results

### 3.1 Cohort characteristics

Of 108 patients assessed, 44 were excluded: 18 underwent reoperation for recurrent glioblastoma, seven were aged <18 years, 10 had impaired consciousness at the postoperative assessment, and 9 underwent biopsy only. No patient was excluded for declining postoperative concurrent chemoradiotherapy. The clinical analysis therefore included 64 patients (**Figure 1A**). Median age was 59.5 years (IQR 54.8–67.0), 40 (62.5%) were male, median KPS was 80 (IQR 80–90), and 39 (60.9%) underwent GTR.

Preoperative MRI records documented peritumoral edema in 45/51 patients (88.2%) and midline shift in 34/57 patients (59.6%). All 64 patients received postoperative radiotherapy with concurrent temozolomide and postoperative antiseizure medication. At data lock, 56 (87.5%) had documented progression and 8 were censored alive without documented progression (**Table 1**).

**Table 1:** Clinical and perioperative characteristics.

Variable	Value
Age, years	59.5 (54.8–67.0)
Male	40/64 (62.5%)
KPS	80 (80–90)
Assessment timing, days after surgery	35 (30–38), median (range)
Peritumoral edema	45/51 (88.2%)
Midline shift	34/57 (59.6%)
Gross-total resection	39/64 (60.9%)
Postoperative intensive care unit admission	31/62 (50.0%)
Postoperative pulmonary infection	18/53 (34.0%)
Deep-vein thrombosis	10/58 (17.2%)
Progression by data lock	56/64 (87.5%)

Values are median (IQR) or *n*/*N* (%); assessment timing is reported as median (range). Denominators vary where data were missing. Data lock: 25 June 2026. All tumors were glioblastoma, IDH-wild-type, CNS WHO grade 4. All 64 patients received postoperative radiotherapy with concurrent temozolomide and postoperative antiseizure medication.

### 3.2 Symptom screening profile

Median GAD-7 was 10.0 (IQR 8.0–10.0): 31 patients (48.4%) had moderate and 2 patients (3.1%) had severe symptoms, yielding 33 screen-positive patients (51.6%). Median PHQ-9 was 11.0 (IQR 10.0–13.0): 43 patients (67.2%) had moderate and 9 patients (14.1%) had moderately severe symptoms, yielding 52 screen-positive patients (81.3%) (**Figures 1B, C**).

Nine patients (14.1%) were negative on both screens, 3 (4.7%) were GAD-7-only positive, 22 (34.4%) were PHQ-9-only positive, and 30 (46.9%) were dual positive (**Figure 1D**). GAD-7 and PHQ-9 scores were positively correlated (Spearman’s ρ = 0.484, *p* < 0.001; **Figure 1E**).

### 3.3 Associations with progression-free survival

In the primary Kaplan–Meier comparisons, median PFS was shorter in patients with GAD-7 scores ≥10 than in those with scores <10 (8.9 vs. 11.1 months; log-rank *p* = 0.042), and in patients with PHQ-9 scores ≥10 than in those with scores <10 (9.0 vs. 14.8 months; *p* = 0.002; **Figures 2A, B**; **Table 2**). Exploratory dual-positivity and descriptive four-category curves are provided in **Supplementary Figure S3**. The four-category display was not subjected to formal hypothesis testing.

**Figure 2:**
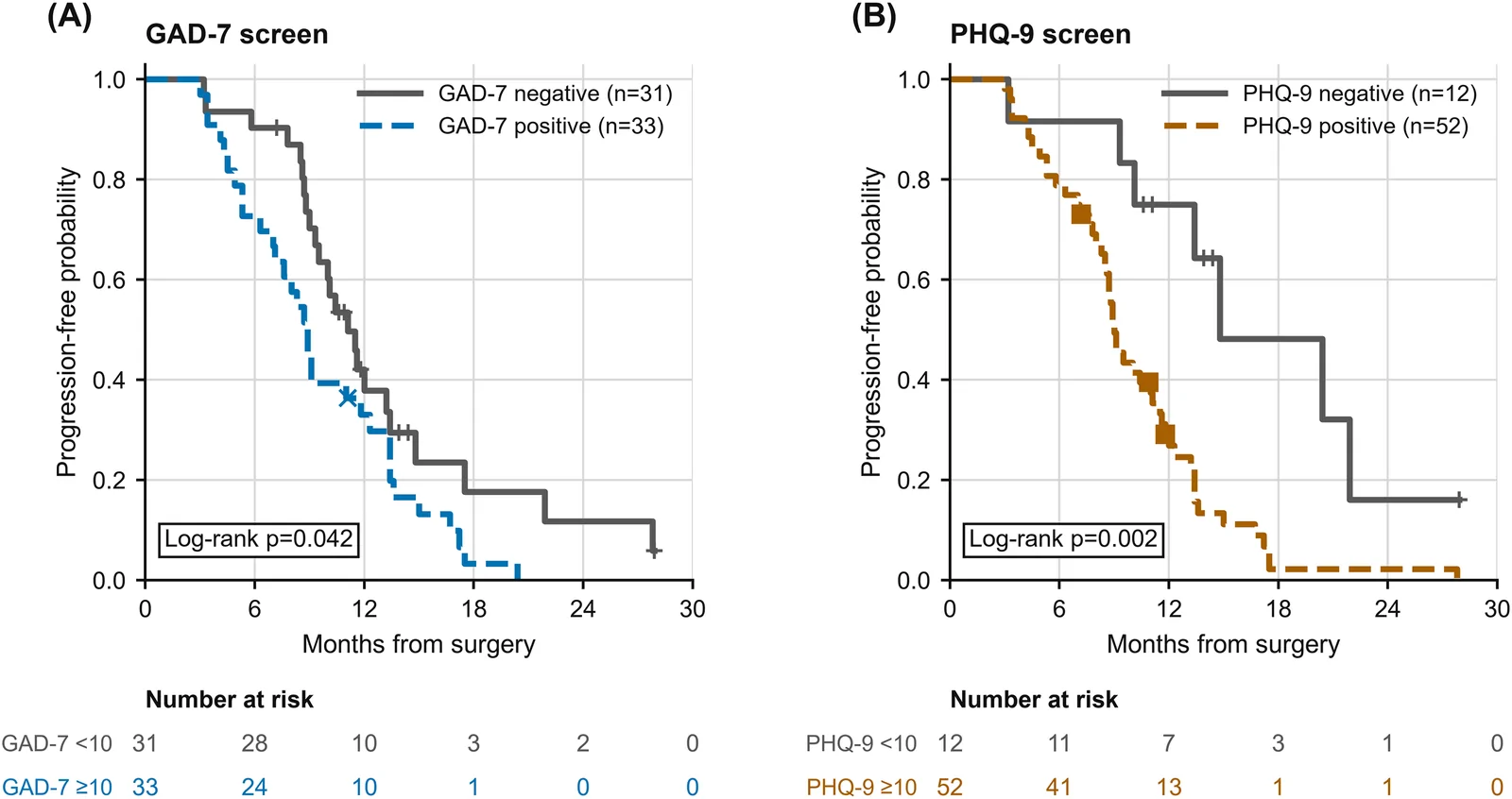
Kaplan–Meier analyses of progression-free survival measured from surgery according to **(A)** GAD-7 and **(B)** PHQ-9 screening status. Positive screens were defined as scores ≥10 and negative screens as scores <10. These were the primary clinical comparisons. *p*-values are from two-sided log-rank tests. Censoring marks and numbers at risk are shown. Exploratory dual-positivity and descriptive four-category curves are provided in **Supplementary Figure S3**.

**Table 2:** Clinical associations with progression-free survival.

A. Kaplan–Meier estimates			
Screen	Groups, *n* (events)	Median PFS, months	Log-rank *p*
GAD-7	Positive 33 (32); negative 31 (24)	8.9 vs. 11.1	0.042
PHQ-9	Positive 52 (49); negative 12 (7)	9.0 vs. 14.8	0.002
Dual positivity (exploratory)	Positive 30 (30); others 34 (26)	8.5 vs. 11.6	0.004

B. Cox regression models			
Measure	Comparison or unit	Univariable HR (95% CI); *p*	Adjusted HR (95% CI); *p*
GAD-7 positivity	≥10 vs. <10	1.76 (1.02–3.05); 0.042	1.92 (1.08–3.42); 0.027
PHQ-9 positivity	≥10 vs. <10	3.47 (1.54–7.82); 0.003	4.22 (1.72–10.35); 0.002
Dual positivity (exploratory)	Both positive vs. others	2.22 (1.28–3.85); 0.004	2.02 (1.14–3.55); 0.015
GAD-7 score (supportive)	Per 1 standard deviation	1.82 (1.27–2.63); 0.001	1.69 (1.20–2.38); 0.003
PHQ-9 score (supportive)	Per 1 standard deviation	1.87 (1.38–2.53); <0.001	1.95 (1.37–2.78); <0.001

Panel A presents Kaplan–Meier estimates and two-sided log-rank tests. Panel B reports separate Cox models. GAD-7 and PHQ-9 screen positivity were the primary comparisons; dual positivity was exploratory, and continuous-score analyses were supportive. Positive screens were defined as scores ≥10, with scores <10 as the reference. Dual positivity required both scores ≥10 and was compared with all other patients. Every adjusted model included the symptom measure, age, KPS, and GTR. Continuous symptom scores were standardized using the sample SD. *n* = 64; 56 progression events. GTR-stratified sensitivity analyses are reported in **Supplementary Table S6**. HR, hazard ratio; CI, confidence interval.

After adjustment for age, KPS, and GTR, GAD-7 positivity (adjusted HR 1.92, 95% CI 1.08–3.42) and PHQ-9 positivity (adjusted HR 4.22, 95% CI 1.72–10.35) were associated with shorter PFS (**Table 2**). Exploratory dual-positivity and supportive continuous-score analyses showed consistent directions of association (**Figure 3**). No statistically significant departures from linearity were detected for age, KPS, or continuous symptom scores. Proportional hazards diagnostics indicated nonproportionality for GTR in the GAD-7 positivity, dual-positivity, and continuous GAD-7 models. GTR-stratified sensitivity analyses yielded similar associations for GAD-7 positivity (HR 1.84, 95% CI 1.03–3.30) and PHQ-9 positivity (HR 3.84, 95% CI 1.53–9.64; **Supplementary Tables S5, S6** and **Supplementary Figures S4–S6**).

**Figure 3:**
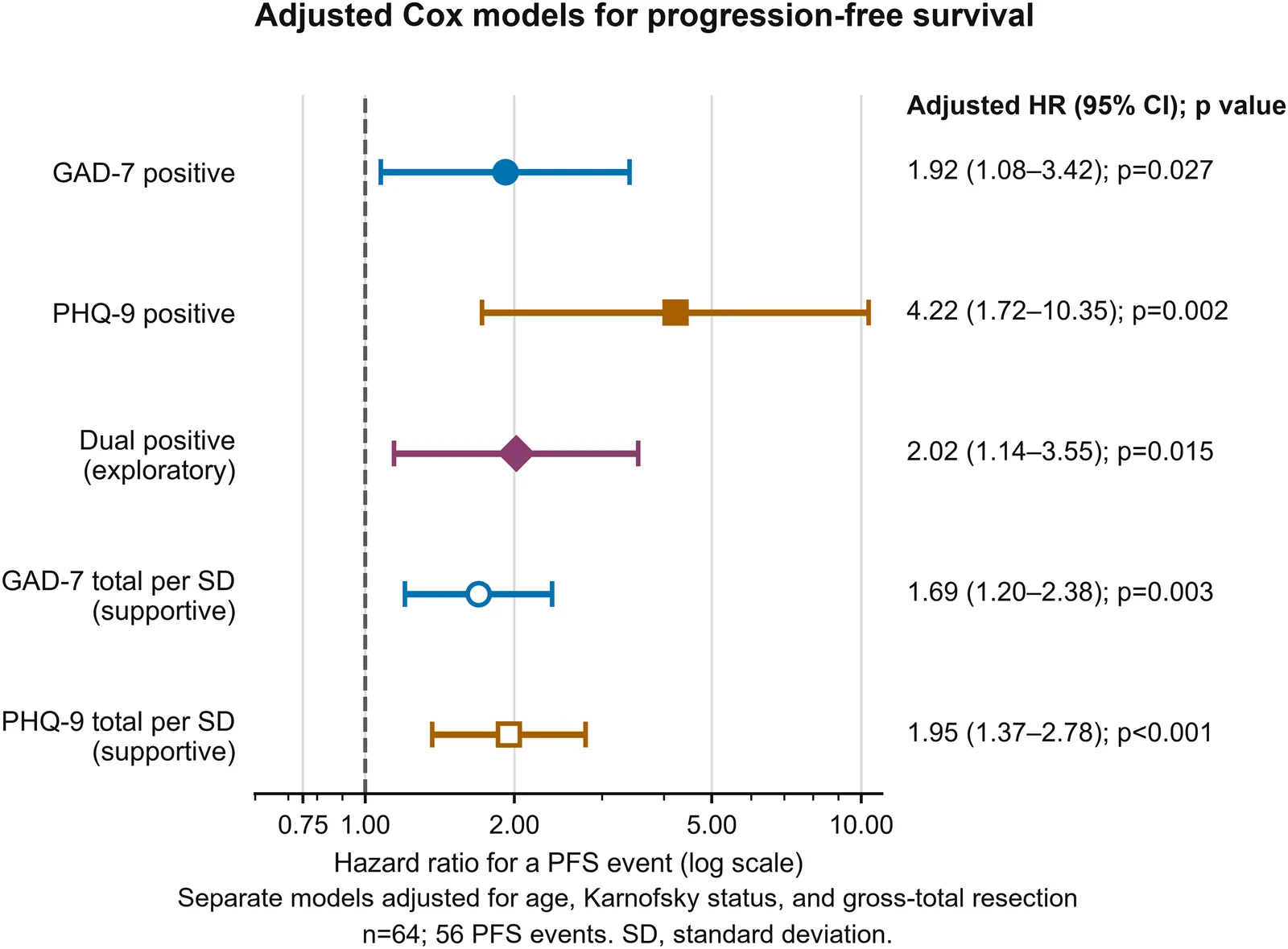
Adjusted associations between postoperative symptom measures and progression-free survival. Each estimate is from a separate Cox model including the symptom measure, age, Karnofsky Performance Status, and gross-total resection. GAD-7 and PHQ-9 positivity were the primary comparisons (scores ≥10 versus <10). Dual positivity was exploratory (both scores ≥10 versus all other patients), and continuous-score analyses were supportive (per one sample standard deviation increase). Points show HRs and lines show 95% CIs. GTR-stratified sensitivity analyses are reported in **Supplementary Table S6**.

### 3.4 Paired GLASS primary–recurrent comparisons

The GLASS analysis included 105 patients with paired presentation and earliest available recurrent profiles (210 RNA profiles); the selected sample was the first recurrence in 96 patients, the second recurrence in 7 patients, and the third recurrence in 2 patients.

Four neurotransmitter-receptor family scores were higher in recurrent than paired primary tumors: adrenergic (Hodges–Lehmann difference +0.208 mean gene-wise *z*-score units, 95% CI 0.092–0.327; BH *q* = 0.006), serotonergic (+0.185, 0.072–0.298; *q* = 0.006), dopaminergic (+0.166, 0.054–0.287; *q* = 0.018), and GABAergic (+0.185, 0.045–0.340; *q* = 0.018) (**Figure 4A**; **Supplementary Table S2**).

**Figure 4:**
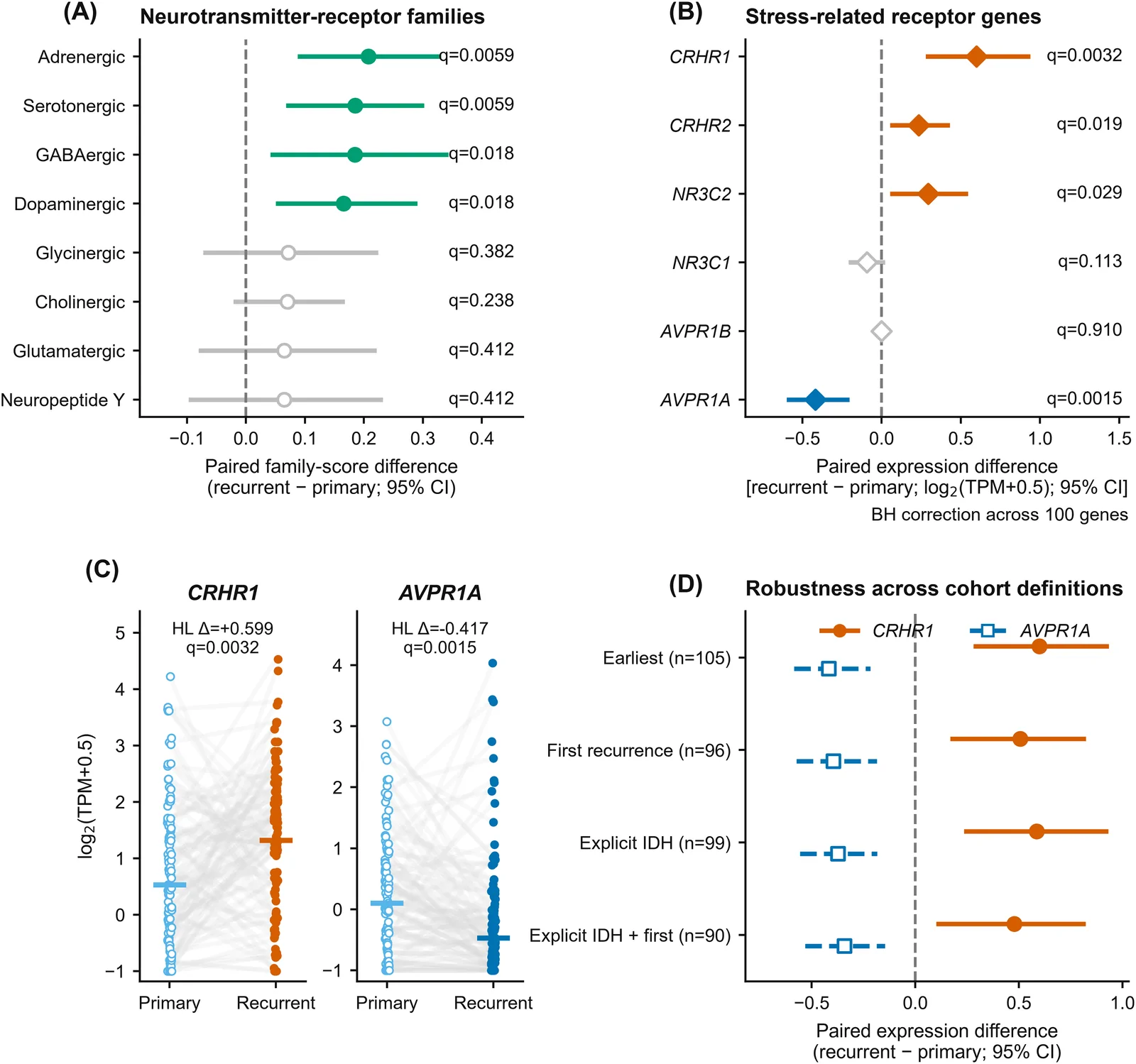
Independent exploratory analysis of receptor-gene expression in paired primary and recurrent GLASS tumors. **(A)** Within-patient differences (recurrent minus primary) in eight neurotransmitter-receptor family scores. Points are Hodges–Lehmann estimates and lines are 95% percentile-bootstrap CIs; filled symbols denote BH *q* < 0.05 across the eight families and open symbols denote BH *q* ≥ 0.05. **(B)** Within-patient expression differences for six stress-related receptor genes. Filled diamonds denote BH *q* < 0.05 and open diamonds denote BH *q* ≥ 0.05 across all 100 genes; direction is shown by *x*-axis position. **(C)** Paired expression values in primary and recurrent tumors for *CRHR1* and *AVPR1A*; horizontal bars show medians. **(D)** *CRHR1* and *AVPR1A* differences across the main and three nested cohort definitions. The main analysis included 105 paired presentation and earliest available recurrent profiles. CIs used 2,000 patient-bootstrap resamples; random seed, 20260723.

Within the exploratory stress-axis extension, *CRHR1* expression was higher in recurrent than in paired primary tumors [Hodges–Lehmann difference +0.599 log2(TPM + 0.5), 95% CI 0.292–0.924; BH *q* = 0.003], whereas *AVPR1A* expression was lower (−0.417, −0.586 to −0.217; *q* = 0.001). *CRHR2* (+0.235, 0.067–0.417; *q* = 0.019) and *NR3C2* (+0.293, 0.068–0.531; *q* = 0.029) were also higher in recurrent tumors in the 105-pair analysis (**Figure 4B, C**; **Supplementary Table S3**).

The *CRHR1* and *AVPR1A* differences retained the same directions and BH *q* < 0.05 in all four nested cohort definitions (*n* = 90–105; **Figure 4D**). After adjustment for the within-patient difference in estimated tumor purity, the paired effects remained +0.581 for *CRHR1* (95% CI 0.289–0.873; BH *q* < 0.001) and −0.375 for *AVPR1A* (−0.607 to −0.144; *q* = 0.006). The adrenergic, serotonergic, dopaminergic, and GABAergic family differences also remained significant after purity adjustment (*q* = 0.003–0.004; **Supplementary Table S4**). Composition associations and the complete 100-gene landscape are provided in **Supplementary Figures S1**, **S2**, respectively.

## 4 Discussion

In this single-center cohort of patients with WHO CNS5-defined IDH-wild-type glioblastoma, greater anxiety and depressive symptom burden at postoperative screening was associated with shorter PFS after adjustment for age, KPS, and GTR. Screening occurred before adjuvant therapy within a defined postoperative window. The separate GLASS analysis identified differences in neurotransmitter-receptor family scores and selected stress-related receptor genes between paired primary and recurrent tumors. These clinical and transcriptomic results address distinct questions and should be interpreted within their respective observational settings.

These findings extend previous reports linking anxiety and depressive symptoms with poorer survival in glioma, although earlier studies varied in tumor grade, molecular classification, assessment instrument, timing, and endpoint (4, 8–10). The present cohort was restricted to WHO CNS5-defined IDH-wild-type glioblastoma and used a defined postoperative assessment window. This molecular and temporal consistency is important when studying symptoms that can fluctuate rapidly around surgery and treatment. GAD-7 and PHQ-9 were selected because they are brief, validated instruments with established cutoffs (17, 18); the concordant binary and continuous analyses support the consistency of the observed associations. The high PHQ-9 screen-positive proportion (81.3%) underscores the importance of assessing depressive symptoms during postoperative recovery. Tumor-related and postoperative symptoms, including fatigue, sleep disturbance, and concentration difficulties, may overlap with some PHQ-9 items, highlighting the need for clinical assessment to distinguish screening positivity from a depressive disorder (23).

In prospective glioma studies, functional impairment has been associated with major depressive disorder (24), while tumor progression has been associated with anxiety and depressive symptoms at follow-up (25). Although symptom assessment in the present study preceded adjuvant therapy, underlying disease activity could already have influenced postoperative symptom scores. GTR status and KPS may not fully capture differences in residual disease or neurological impairment. Reverse causation and residual confounding therefore remain possible, and the observed associations should not be interpreted as causal effects.

Postoperative screening can help identify patients who need further assessment and supportive care. Neuro-oncologists can incorporate patient-completed GAD-7 and PHQ-9 questionnaires into routine consultations, review patients’ responses, and undertake initial clinical assessment as part of standard care. A positive result can prompt assessment of symptom severity and safety alongside neurological status, cognition, sleep, pain, medication use, functional recovery, and family support. Clinical practice guidelines recommend validated screening followed by assessment and treatment matched to symptom severity and patient preference (6, 7). This supports a stepped approach ranging from information, monitoring, and rehabilitation support to psycho-oncology or psychiatric assessment. Evidence-based psychotherapy and, when clinically indicated, pharmacotherapy can help reduce anxiety and depressive symptoms. Treatment should aim for symptom remission or reduction to minimal levels where feasible, alongside improved daily functioning and quality of life; repeated GAD-7 and PHQ-9 scores can help monitor response when interpreted alongside clinical evaluation (6, 7). A scoping review identified potential benefits of psychotherapy for depressive symptoms and quality of life in adults with brain tumors, while emphasizing the need for a stronger disease-specific evidence base (26). Prospective interventional studies could examine whether symptom management influences PFS.

Anxiety and depressive symptoms can change over time, supporting repeated assessment after the initial postoperative screen. In a prospective high-grade glioma cohort, distress followed distinct trajectories during the first 6 months after chemoradiotherapy began, with supportive-care needs changing in parallel (27). Reassessment during concurrent treatment, adjuvant temozolomide, surveillance, and recurrence would therefore be clinically informative. At transfer of care, neurosurgeons should communicate available screening results and the need for ongoing anxiety and depressive symptom monitoring to the patient’s general practitioner and treating oncologist. Follow-up should be coordinated between primary care and oncology, with in-person assessment when clinically appropriate and feasible. A randomized trial of the brain-tumor-specific Tele-MAST program found lower depressive symptoms after a tailored telehealth intervention than after standard care in distressed adults with primary brain tumors, including patients with high-grade glioma (28). Telehealth-based psychological support may complement ongoing clinical care when appropriate, while in-person assessment remains important for comprehensive evaluation. This evidence supports timely referral for symptom relief and daily functioning and provides a basis for trials that integrate psychological care into glioblastoma treatment pathways.

The GLASS analysis addressed a separate exploratory question concerning neurotransmitter- and stress-related receptor expression in paired primary and recurrent tumors. The within-patient design reduced between-patient variability in this molecular comparison. However, the GLASS and clinical cohorts were independent, and no patient-level data linked anxiety or depressive symptoms to receptor expression. The observed expression differences therefore cannot be attributed to symptom status or interpreted as a mechanism underlying the clinical association with PFS. These findings are hypothesis-generating and identify candidate receptor systems for future studies with linked symptom and molecular measurements.

Experimental studies provide a rationale for investigating stress-responsive and neuronal receptor systems in glioma. Stress-responsive signaling can influence immune, inflammatory, angiogenic, and microenvironmental processes across tumor contexts (13–15). In glioblastoma models, chronic stress accelerated growth through dopamine–*DRD2* signaling (11), while glutamatergic neuron-to-glioma synapses promoted tumor progression (12). Animal studies also link corticotropin-releasing hormone, mineralocorticoid, and vasopressin receptor systems to stress responses and anxiety-like behavior (29–32). The receptor-expression differences observed in GLASS therefore identify candidates for further functional, single-cell, and spatial investigation. However, bulk transcript abundance does not establish receptor activity and may reflect treatment exposure, tumor evolution, or changes in cellular composition (20). Purity adjustment and nested-cohort analyses support the consistency of these expression findings within GLASS.

Related research has examined serotonergic biology and antidepressant repurposing in glioblastoma. Wu et al. reported that preoperative antidepressant use was associated with lower intratumoral serotonin levels, while serotonin-pathway metabolites showed no clear association with survival (33). A small phase Ib/IIa study evaluated the safety and feasibility of CUSP9v3, a nine-drug regimen including sertraline, combined with temozolomide in recurrent glioblastoma (34). Network and molecular docking analyses have also proposed candidate targets for vortioxetine in glioblastoma (35). These studies provide context for further investigation but do not establish that alleviating anxiety or depressive symptoms improves tumor control.

Strengths include the molecularly defined clinical cohort, a specified postoperative assessment window, two validated symptom scales, consistent covariate adjustment, and paired primary–recurrent transcriptomic comparisons within GLASS. The clinical sample was nevertheless small and selected from patients able to complete postoperative screening and receive chemoradiotherapy. The high proportion of progression events does not eliminate imprecision from the small and imbalanced screening groups, particularly the PHQ-9-negative reference group (*n* = 12). Retrospective progression ascertainment without independent centralized imaging review may have introduced variability in recorded timing. A major limitation is that *MGMT* promoter methylation data were incomplete and corticosteroid exposure was not recorded; neither variable was included in the models. Residual confounding, including possible differences in antiseizure medication regimens, remains possible.

Future multicenter studies should combine repeated symptom assessment with standardized imaging, treatment data, and matched biospecimens from the same patients. Such a design could evaluate the clinical associations, clarify symptom trajectories, and examine the relevance of the receptor pathways prioritized by the paired GLASS comparison. Functional studies would be needed to investigate any proposed mechanistic links.

## 5 Conclusion

Greater postoperative anxiety and depressive symptom burden was associated with shorter PFS in patients with IDH-wild-type glioblastoma assessed before adjuvant therapy. These findings support attention to anxiety and depressive symptoms during postoperative care, without establishing a causal effect on disease progression. The independent GLASS analysis identified receptor-expression differences between primary and recurrent tumors that are hypothesis-generating. Prospective studies incorporating repeated symptom assessments, more complete clinical data, and matched molecular sampling are needed to evaluate these observations.

## Data Availability

The datasets presented in this study can be found in online repositories. The names of the repository/repositories and accession number(s) can be found in the article/**Supplementary Material**.
