# Supplementary material for: Association of postoperative anxiety and depressive symptom severity with progression-free survival in IDH-wild-type glioblastoma
Source: Front Oncol. 2026 Oct 19;16:1949803. doi: 10.3389/fonc.2026.1949803 (PMC13724391; doi:10.3389/fonc.2026.1949803)
Supplement: Supplementary file 1 [file DataSheet1.docx]

Supplementary Material

Association of postoperative anxiety and depressive symptom severity with progression-free survival in IDH-wild-type glioblastoma

# Supplementary Methods

## GLASS cohort and panel construction

Both samples in each pair were required to have deposited annotations of glioblastoma and histological grade IV, with IDH-wild-type status recorded as IDH_STATUS = IDHwt or an IDH_CODEL_STATUS value beginning with IDHwt. Selection relied on deposited histopathological and molecular annotations, without independent WHO CNS5 reclassification. GLASS cases linked to the TCGA project were excluded during cohort construction; the resulting cohort comprised 105 patients, including 96 first, seven second, and two third recurrences. Kallisto TPM values were transformed as log2(TPM+0.5). Ninety-four receptor genes were assigned to eight neurotransmitter families. The exploratory stress-related extension added *CRHR1*, *CRHR2*, *NR3C1*, *NR3C2*, *AVPR1A*, and *AVPR1B*, yielding 100 genes. For the main family analysis, each gene was standardized across the 210 paired profiles and family scores were means of member-gene z scores. The within-patient difference was defined as recurrent minus primary. Supplementary Data S1 records all gene-to-family assignments.

## Paired inference

Family-score and individual-gene differences were tested using two-sided Wilcoxon signed-rank tests. Effects are reported as Hodges–Lehmann paired-difference estimates with 95% percentile CIs from 2,000 patient-bootstrap resamples. Benjamini–Hochberg correction was applied across eight family tests and separately across all 100 individual-gene tests within each cohort. Rank-biserial correlations and the number and percentage of patients with higher expression in recurrent tumors are provided in the source tables.

## Robustness analyses

Nested subsets restricted the main cohort to first recurrence (n=96), explicit IDH_STATUS annotation (n=99), and their intersection (n=90). Purity adjustment used ordinary least-squares models of the within-patient expression or family-score difference on the corresponding difference in ESTIMATE-derived tumor purity, with HC3 robust covariance. Exploratory composition associations used within-patient differences in immune, stromal, and estimated-purity scores. Analyses used Python 3.12.13, NumPy 2.3.5, pandas 3.0.1, SciPy 1.18.0, statsmodels 0.14.6, and random seed 20260723.

## Accompanying supplementary tables and data

Supplementary Tables S1–S4, Supplementary Data S1, and the GLASS cohort manifest are supplied in the accompanying workbook Supplementary_Tables_and_Data.xlsx. Tables S5–S6 and Figures S1–S6 appear in this document.

Supplementary Table S1. Cohort definition, sample composition, and nested sensitivity subsets for the paired GLASS transcriptomic cohort.

Supplementary Table S2. Eight neurotransmitter-receptor family effects in the main and sensitivity cohorts.

Supplementary Table S3. Individual-gene effects for all 100 receptor genes in the main and sensitivity cohorts.

Supplementary Table S4. Nested-cohort and tumor-purity robustness analyses.

Supplementary Data S1. Assignment of the 100 genes to neurotransmitter-receptor families or the exploratory stress-related extension.

Supplementary Cohort Manifest. Patient-level manifest for the 105-pair GLASS cohort and nested eligibility subsets.

# Supplementary Figure S1 Associations with composition scores

Associations with ESTIMATE-derived composition scores. Marker area increases with the absolute Spearman correlation between within-patient family-score differences and differences in immune score, stromal score, or estimated purity. Filled circles denote positive correlations and open squares denote negative correlations. Asterisks indicate Benjamini–Hochberg q<0.05 across all 24 associations. Both variables were derived from the same bulk-RNA profiles.

# Supplementary Figure S2 Receptor gene expression landscape

Effect and significance landscape for all 100 receptor genes. The x-axis shows the Hodges–Lehmann paired recurrent-minus-primary expression difference and the y-axis shows −log10 of the Benjamini–Hochberg q value across 100 genes. Filled circles denote q<0.05, open circles denote q≥0.05, and diamonds identify the six stress-related genes. Selected genes are labeled.

# Supplementary Figure S3 Combined screening phenotypes and PFS

Exploratory and descriptive analyses of progression-free survival according to combined screening status. (A) Dual screen positivity versus all other patients, presented as an exploratory comparison. (B) Four-category screening phenotype, presented descriptively because of the small subgroup sizes. No formal hypothesis tests were performed across the four categories. Positive screens were defined as scores ≥10. Censoring marks and numbers at risk are shown.

# Cox model diagnostics and sensitivity analyses

These analyses evaluated model assumptions and the sensitivity of the associations between postoperative anxiety or depressive symptom scores and progression-free survival in 64 patients with IDH-wild-type glioblastoma, including 56 progression events.

Five separate Cox models included the relevant symptom measure, age, Karnofsky Performance Status (KPS), and gross-total resection (GTR), using Efron handling of tied event times. Proportional hazards (PH) were assessed using scaled Schoenfeld residuals, covariate-specific and global tests with the Kaplan–Meier time transformation, and graphical inspection. Nonlinearity was assessed using likelihood ratio tests comparing each linear term with a natural cubic spline. Analyses used R 4.6.0 and survival 3.8.6.

## Supplementary Table S5 Diagnostics for the adjusted Cox models

## A Proportional hazards tests

| **Symptom measure** | **Symptom P** | **Age P** | **KPS P** | **GTR P** | **Global P** |
| --- | --- | --- | --- | --- | --- |
| GAD-7 positivity (primary) | 0.736 | 0.221 | 0.848 | 0.015 | 0.137 |
| PHQ-9 positivity (primary) | 0.658 | 0.254 | 0.723 | 0.080 | 0.432 |
| Dual positivity (exploratory) | 0.792 | 0.152 | 0.940 | 0.017 | 0.160 |
| GAD-7 score per SD (supportive) | 0.996 | 0.386 | 0.915 | 0.043 | 0.333 |
| PHQ-9 score per SD (supportive) | 0.732 | 0.456 | 0.850 | 0.051 | 0.301 |

## B Tests for departure from linearity

| **Symptom measure** | **Age P** | **KPS P** | **Symptom score P** |
| --- | --- | --- | --- |
| GAD-7 positivity (primary) | 0.365 | 0.535 | Not applicable |
| PHQ-9 positivity (primary) | 0.657 | 0.634 | Not applicable |
| Dual positivity (exploratory) | 0.218 | 0.331 | Not applicable |
| GAD-7 score per SD (supportive) | 0.477 | 0.559 | 0.083 |
| PHQ-9 score per SD (supportive) | 0.475 | 0.455 | 0.821 |

Notes. All values are P values. Screen positivity was defined as a score ≥10 on the respective instrument, with scores <10 as the reference. Dual positivity required both scores ≥10 and was compared with all other patients. Each spline used two degrees of freedom, with one internal knot at the median and boundary knots at the observed minimum and maximum. One term was replaced at a time, so each likelihood ratio test had one additional degree of freedom. Binary symptom measures were not subjected to a linearity test. Continuous scores were standardized by the sample standard deviation (SD). GAD-7, Generalized Anxiety Disorder-7; PHQ-9, Patient Health Questionnaire-9.

# Supplementary Table S6 Sensitivity analyses stratified by GTR

## A Associations with progression-free survival

| **Symptom measure** | **GTR-adjusted HR (95% CI)** | **P** | **GTR-stratified HR (95% CI)** | **P** |
| --- | --- | --- | --- | --- |
| GAD-7 positivity (primary) | 1.92 (1.08–3.42) | 0.027 | 1.84 (1.03–3.30) | 0.040 |
| PHQ-9 positivity (primary) | 4.22 (1.72–10.35) | 0.002 | 3.84 (1.53–9.64) | 0.004 |
| Dual positivity (exploratory) | 2.02 (1.14–3.55) | 0.015 | 1.91 (1.08–3.40) | 0.027 |
| GAD-7 score per SD (supportive) | 1.69 (1.20–2.38) | 0.003 | 1.56 (1.11–2.20) | 0.011 |
| PHQ-9 score per SD (supportive) | 1.95 (1.37–2.78) | <0.001 | 1.78 (1.25–2.54) | 0.001 |

## B Proportional hazards tests in the GTR stratified models

| **Symptom measure** | **Symptom P** | **Age P** | **KPS P** | **Global P** |
| --- | --- | --- | --- | --- |
| GAD-7 positivity (primary) | 0.446 | 0.344 | 0.436 | 0.720 |
| PHQ-9 positivity (primary) | 0.641 | 0.201 | 0.946 | 0.553 |
| Dual positivity (exploratory) | 0.860 | 0.243 | 0.481 | 0.685 |
| GAD-7 score per SD (supportive) | 0.643 | 0.479 | 0.543 | 0.832 |
| PHQ-9 score per SD (supportive) | 0.378 | 0.454 | 0.669 | 0.692 |

Notes. All models included 64 patients and 56 progression events. The GTR-adjusted models included the symptom measure, age, KPS, and GTR as covariates. The sensitivity models retained the symptom measure, age, and KPS and allowed separate baseline hazard functions for GTR and non-GTR patients. GTR was a stratification factor and was not assigned a separate HR. These analyses used the full cohort rather than fitting separate subgroup models. PH tests used scaled Schoenfeld residuals with the Kaplan–Meier time transformation. HR, hazard ratio; CI, confidence interval; other abbreviations are defined in Table S5.

The primary symptom associations remained similar after stratification by GTR. The term-specific and global PH tests in the stratified models did not detect statistically significant departures from proportional hazards.

# Supplementary Figure S4 GTR proportional hazards diagnostics

Scaled Schoenfeld residual diagnostics for GTR in the five adjusted Cox models. Panels A–E correspond to GAD-7 positivity, PHQ-9 positivity, dual positivity, continuous GAD-7 score, and continuous PHQ-9 score. Solid black curves show smoothed time-varying coefficients, dashed curves show approximate 95% confidence limits, and blue dotted lines show the fitted constant coefficients. The horizontal axis uses the Kaplan–Meier time transformation, labeled in months from surgery. The displayed P values test PH for GTR.

# Supplementary Figure S5 Continuous variable functional forms

Panels A and B show age and KPS in the GAD-7 positivity model; panels C and D show age and KPS in the PHQ-9 positivity model. Panels E and F show GAD-7 and PHQ-9 scores in their respective continuous-score models. Blue curves represent two-degree-of-freedom natural cubic splines, red dashed curves represent linear terms, and shaded bands show pointwise 95% confidence intervals for the spline estimates. Effects are expressed as log HR relative to the median, and rug marks indicate observed values. P values are from likelihood ratio tests for nonlinearity. Other covariates were retained as in the corresponding adjusted model.

# Supplementary Figure S6 Symptom proportional hazards diagnostics after GTR stratification

Scaled Schoenfeld residual diagnostics for the symptom measure in each GTR-stratified model. Panels A–E correspond to GAD-7 positivity, PHQ-9 positivity, dual positivity, continuous GAD-7 score, and continuous PHQ-9 score. All models retained adjustment for age and KPS. Solid black curves show smoothed time-varying coefficients, dashed curves show approximate 95% confidence limits, and blue dotted lines show fitted constant coefficients. The horizontal axis uses the Kaplan–Meier time transformation, labeled in months from surgery. The displayed P values test PH for the symptom measure; covariate-specific and global tests are reported in Table S6B.
